# Supplementary material for: Content-rich biological network constructed by mining PubMed abstracts
Source: BMC Bioinformatics. 2004 Oct 8;5:147. doi: 10.1186/1471-2105-5-147 (PMC528731; doi:10.1186/1471-2105-5-147)
Supplement: Additional File 5 — The original Chilibot query results of the term "long-term potentiation (LTP)" and 22 other terms, limiting the latest references analyzed to the years 1990, 1995, 2000, and 2004. [file 1471-2105-5-147-S5.bz2 › chilibotAdditionalFile5/ltp2004/html/PLC_TRKB.html]

 


 **PLC** and **TRKB** 
  
Found 15 abstracts in PubMed,  **15 abstracts were retrieved and analyzed**.  


---

 Search Google  |
 PDF files only 
|  EDU domain only 

---

**Interactive relationship** (e.g. stimulation, inhibition, etc)

- These results suggest that two different pathways, the c ras and the  **PLC**  gamma pathway, are activated by  **TrkB**  receptors in primary neurons.  Ref: 7595513 J Neurochem, 1995
- SIN 1 treatment induced tyrosine phosphorylation of phospholipase C gamma1  **PLC**  gamma1 and induced its binding with activated  **TrkB** , similar to that seen with BDNF downstream signaling pathways.  Ref: 11095525 Neuroreport, 2000
- BDNF and NT 4 5 also induced an autophosphorylation of  **TrkB**  receptors and subsequently resulted in a phosphorylation and binding of phospholipase C gamma  **PLC**  gamma and SH2 containing sequence to the autophosphorylated  **TrkB**  receptors.  Ref: 7595513 J Neurochem, 1995
- Activation of  **TrkB**  by brain derived nerve growth factor BDNF led to production of a  **PLC**  dependent, nonselective cation conductance in pontine neurons.  Ref: 10677043 Neuron, 1999
- ...  **TrkB**  mutated at the  **PLC**  gamma1 binding site supported growth and survival well.  Ref: 10985347 Neuron, 2000
- Using immunoprecipitation, PI3 kinase and tyrosine phosphorylated  **PLC**  gamma1 and SHC were shown to be associated with tyrosine phosphorylated  **TrkB**  in response to both BDNF and NT 4.  Ref: 10486198 Exp Neurol, 1999

**Parallel relationship** (e.g. studied together, co-existance, homology, etc.)

- Thus, activation of  **TrkB**  and  **PLC**  leads to a TRPC3 dependent cation influx in CNS neurons.  Ref: 10677043 Neuron, 1999
- We suggest that sustained activation of Trk B  [ **TRKB** ]  receptor tyrosine kinase and  **PLC**  gamma occurs after a brief BDNF application and contributes to the short term maintenance <.  Ref: 12960768 Neuroreport, 2003
- Our results demonstrate that the signal transduction pathways through TRK A and TRK B  [ **TRKB** ]  in MP N TS cells are functional and similar, and the main downstream signaling pathways from the three neurotrophins are mitogen activated protein kinase cascades through Shc, activated Ras, ERK 1 and ERK 2, and the transduction pathway through  **PLC**  gamma1.  Ref: 11223544 Jpn J Cancer Res, 2001
- BDNF increased intracellular calcium through the  **PLC**  gamma phosphorylation after the  **TrkB**  receptor tyrosine kinase activation.  Ref: 12960768 Neuroreport, 2003
- Last, it was revealed that the enhancement of glutamate release by BDNF was dependent on the  **TrkB**   **PLC**  gamma pathway.  Ref: 11701755 J Neurochem, 2001
